# Altered expression of Sialyl Lewis X in experimental models of Parkinson’s disease

**DOI:** 10.1007/s00109-023-02415-3

**Published:** 2024-01-10

**Authors:** Maria João Nunes, Andreia Neves Carvalho, Alexandra I. Rosa, Paula A. Videira, Maria João Gama, Elsa Rodrigues, Margarida Castro-Caldas

**Affiliations:** 1 Research Institute for Medicines (iMed.ULisboa), Faculty of Pharmacy, Universidade de Lisboa, Av. Prof. Gama Pinto, 1649-003 Lisbon, Portugal; 2 Department of Life Sciences, UCIBIO, NOVA School of Science and Technology, Universidade NOVA de Lisboa, 2829-516 Caparica, Portugal; 3 CDG & Allies - Professionals and Patient Associations International Network (CDG & Allies - PPAIN), NOVA School of Science and Technology, Universidade NOVA de Lisboa, 2829-516 Caparica, Portugal

**Keywords:** Glycosylation, MPTP, Parkinson’s disease, Sialyl Lewis X

## Abstract

**Abstract:**

The mechanisms underlying neurodegeneration in Parkinson’s disease (PD) are still not fully understood. Glycosylation is an important post-translational modification that affects protein function, cell-cell contacts and inflammation and can be modified in pathologic conditions. Although the involvement of aberrant glycosylation has been proposed for PD, the knowledge of the diversity of glycans and their role in PD is still minimal. Sialyl Lewis X (sLeX) is a sialylated and fucosylated tetrasaccharide with essential roles in cell-to-cell recognition processes. Pathological conditions and pro-inflammatory mediators can up-regulate sLeX expression on cell surfaces, which has important consequences in intracellular signalling and immune function. Here, we investigated the expression of this glycan using in vivo and in vitro models of PD. We show the activation of deleterious glycation-related pathways in mouse striatum upon treatment with 1-methyl-4-phenyl-1,2,3,6-tetrahydropyridine (MPTP), a toxin-based model of PD. Importantly, our results show that MPTP triggers the presentation of more proteins decorated with sLeX in mouse cortex and striatum in a time-dependent manner, as well as increased mRNA expression of its rate-limiting enzyme fucosyltransferase 7. sLeX is expressed in neurons, including dopaminergic neurons, and microglia. Although the underlying mechanism that drives increased sLeX epitopes, the nature of the protein scaffolds and their functional importance in PD remain unknown, our data suggest for the first time that sLeX in the brain may have a role in neuronal signalling and immunomodulation in pathological conditions.

**Key messages:**

- MPTP triggers the presentation of proteins decorated with sLeX in mouse brain.
- MPTP triggers the expression of sLeX rate-limiting enzyme FUT 7 in striatum.
- sLeX is expressed in neurons, including dopaminergic neurons, and microglia.
- sLeX in the brain may have a role in neuronal signalling and immunomodulation.

**Supplementary Information:**

The online version contains supplementary material available at 10.1007/s00109-023-02415-3.

## Introduction

The mechanistic pathways associated with neurodegeneration in Parkinson’s disease (PD) are still not fully understood but are known to involve oxidative stress, mitochondrial dysfunction and neuroinflammation [1]. Activation of these deleterious mechanisms is associated with post-translational modifications, namely glycation and aberrant glycosylation, decreased levels of endogenous scavengers and impaired quality control systems, leading to the accumulation of dysfunctional proteins [2] that further contribute to the mechanisms underlying dopaminergic cell death in PD. The observation of reactive microglia and pro-inflammatory markers in PD patients’ brains [3], a phenotype which others and we showed to be recapitulated in experimental PD models [3–5], further supports the interplay between neuroinflammation and neurodegeneration.

Microglia swiftly respond to environmental stimuli, but in a context of chronic injury, such as in neurodegenerative diseases, microglia can sustain and exacerbate the inflammatory response contributing to neuronal damage. Microglia activation is highly regulated by glycans expressed on the surface of neighbouring cells, as microglia express a multitude of lectins, including selectins able to recognize normal or altered glycocalyx to which microglia promptly respond [6]. Thus, aberrant glycosylation can alter microglia function and exacerbate neuroinflammation [6]. Indeed, glycosylation has recently been acknowledged to play a role in neurodegenerative diseases [7–9]. Yet, the current understanding of the heterogeneity and functions of glycans in the brain is very limited, particularly in PD. A previous study shows increased overall sialylation in the striatum of PD patients [10], indicating that aberrant glycosylation may correlate with neurodegeneration, but the pathways involved are still not completely understood.

**Fig. 1:**
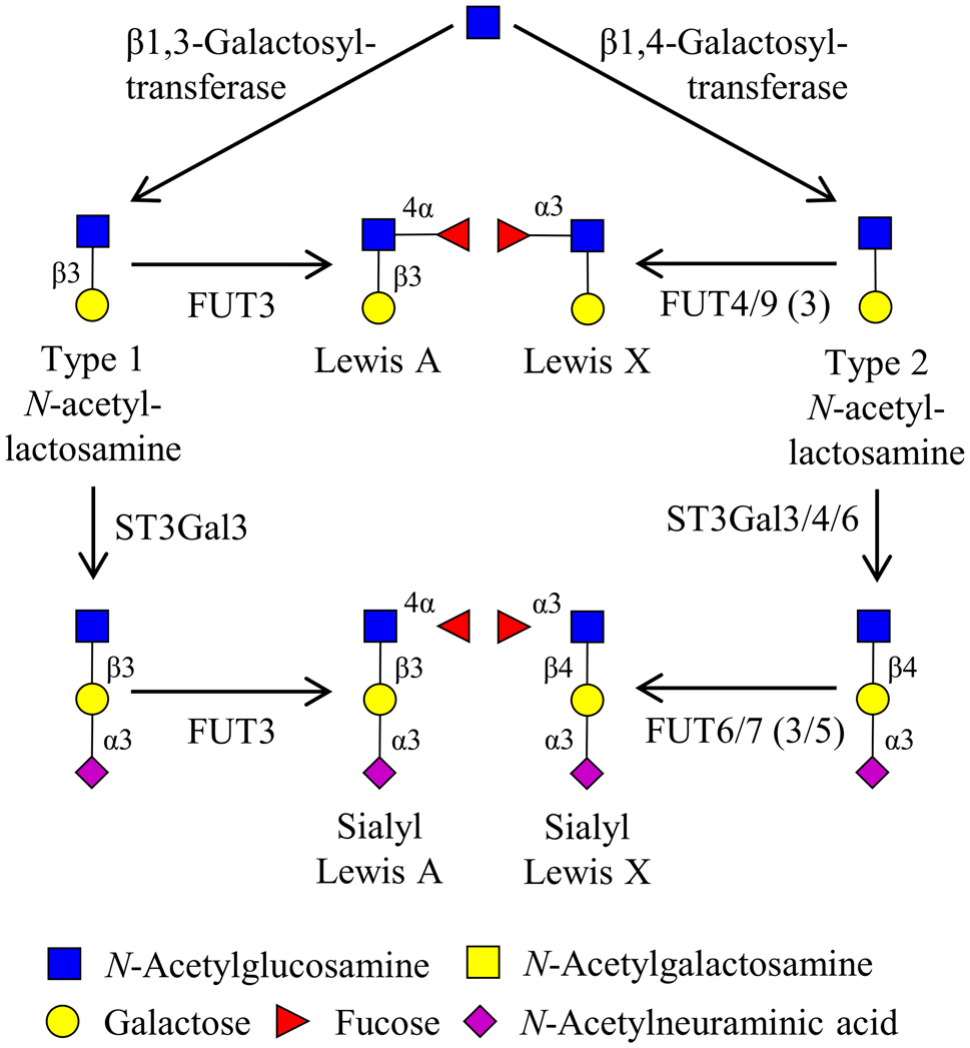
Structure and biosynthesis of sLeX and Lex. Anomers, linkage positions and enzymes involved in the reactions are indicated. sLeX is a tetrasaccharide composed of a sialic acid, fucose and an *N*-acetyllactosamine. In humans, according to the figure, sLeX is synthesized by four fucosyltransferases: FUT3, FUT5, FUT6 and FUT7. The other three enzymes of the α2,3(*N*)-sialyltransferase (ST3GAL) family ST3GAL3, ST3GAL4 and ST3GAL6 participate in the synthesis of the sLeX precursor. All enzymes known to be able to perform a reaction are listed; note that only some of them are expressed in mouse tissues, namely FUT4, FUT7 and FUT9. Monosaccharides are depicted in the figure legend

Sialyl Lewis X (sLeX or CD15s, NeuAc-α (2,3)-Gal-β (1,4)-[Fuc-α (1,3)]-GlcNAc-R) is a glycan with a critical role in inflammation and overexpressed in pathological conditions. It mediates intercellular adhesion and intracellular signalling by interacting with selectins [11, 12]. The expression of sLeX on the surface of leukocytes and binding to selectins is a prerequisite for their extravasation [13, 14]. The biosynthesis of sLeX is catalyzed by α(1,3)-fucosyltransferases (FUTs), which constitute a family of six Golgi isoenzymes, FUT3, FUT4, FUT5, FUT6, FUT7 and FUT9 [12, 15] (Fig. 1). Several reports indicate that in humans, biosynthesis of sLeX is mainly driven by FUT7 and FUT6, and to a lesser extent by FUT3 and FUT5 (Fig. 1). Thus, the up-regulation of one of these enzymes promotes sLeX expression [12], whereas the knockdown was shown to inhibit the synthesis of sLeX in different cells [12]. However, mice have only three functional homologs, Fut4, Fut7 and Fut9 [15]. Of those, the enzyme FUT7 is the sole responsible for the synthesis of sLeX (CD15s), while FUT4 and FUT9 can biosynthesize LeX (CD15), but not sLeX [12, 15] (Fig. 1).

Under physiological conditions, sLeX is expressed at very low levels. The expression of the fucosyltransferases and subsequent sLeX levels increase in response to pro-inflammatory cytokines, giving sLeX a critical role in cell-to-cell recognition in immune processes [2, 11, 16]. Moreover, the variety of protein scaffolds carrying the sLeX epitope contributes to diversifying the presentation and accessibility of this glycan to selectins. In addition, the intracellular signalling associated with a certain cell surface protein can be modified by these additional glycan-protein interactions [17, 18].

Although sLeX was suggested to mediate recognition between different cell types in the central nervous system, the literature regarding sLeX expression in brain cells is very scarce. sLeX in leukocytes mediates the extravasation through the blood–brain barrier [11, 19], and its expression was described in cultured human microglia [11, 20] and on *O*-mannose in whole mouse brain [21], but the functional significance of this epitope in the brain is still unknown.

In the present work, we aimed to study the expression of sLeX in mouse brain and to understand its modulation in PD models. For that, we used a sub-acute 1-methyl-4-phenyl-1,2,3,6-tetrahydropyridine (MPTP) mouse model of PD. We also used primary mouse neurons treated with the active toxic metabolite of MPTP, 1-methyl-4-phenylpyridinium iodide (MPP^+^). Glycation-related pathways were evaluated as a measure of MPTP toxicity. We show for the first time the presentation of more proteins decorated with sLeX in mouse cortex and striatum, and up-regulation of sLeX rate-limiting enzyme FUT7 expression after MPTP administration. We also show that sLeX is expressed in neurons, including dopaminergic neurons, and microglia. Although the functional importance of sLeX in PD remains unknown, our data unravels new modifications of the glycosylation pattern in parkinsonian mouse brain.

## Material and methods

### Ethics statement

All procedures were conducted in accordance with European regulations (European Union Directive 2010/63/EU). Animal facilities and the people directly involved in animal experiments were certified by the Portuguese regulatory entity—Direção Geral de Alimentação e Veterinária.

### Animals and treatments

Twelve-week-old male C57BL/6 wild-type mice were maintained under standard laboratory conditions with free access to a standard diet and water. Mice were intraperitoneally (i.p.) injected with MPTP (Sigma-Aldrich), in a single dose of 40 mg/kg body weight, or saline (control). After 1 h, 3 h, 6 h or 52 days post-MPTP administration, mice were deep anesthetized with a mixture of ketamine hydrochloride (150 mg/kg) plus medetomidine (0.3 mg/kg) and sacrificed by exsanguination perfusion with saline. Brains were collected and processed for cryostat slicing or dissected as previously described [5, 22] to remove the striatum and the cerebral cortex at the level of the nigrostriatal axis (Bregma − 3.20 to 1.00), for protein and RNA extraction.

### Cell culture conditions and cell treatments

Primary cultures of C57BL/6 mouse cortical neurons were prepared from 17- to 18-day-old foetuses, as described previously [4]. After 15 days in culture, cells were treated with 0.5 mM MPP^+^ (Sigma-Aldrich), the toxic metabolite of MPTP, for 3 h. Controls consisted of treating the cells with vehicle.

### Western blot analysis

Protein extracts were prepared from mouse brain or primary cortical neurons as previously described [4, 5]. Protein extracts from brain tissue (50 µg) and primary neurons (30 µg) were subjected to SDS-PAGE gels, electroblotted onto Immobilon P membrane (Millipore, Bedford, MA, USA), and incubated with specific antibodies against S100β (sc-136061, Santa Cruz Biotech.), receptor for advanced glycation end-products (RAGE, sc-365154, Santa Cruz Biotech.), glyoxalase 1 (sc-133214, Santa Cruz Biotech.), interleukin-1β (IL-1β, sc-12742, Santa Cruz Biotech.), tumour necrosis factor-α (TNF-α, 500-P64, Peprotech) or sLeX (CD15s, 551,344, BD Pharmingen), followed by incubation with the specific horseradish peroxidase–conjugated anti-mouse or anti-rabbit (Bio-Rad Lab.) secondary antibodies. The immunocomplexes were visualized by chemiluminescent detection in a ChemiDoc™ MP imaging system from Bio-Rad Laboratories. Staining with anti-glyceraldehyde 3-phosphate dehydrogenase (GAPDH, sc-32233, Santa Cruz Biotech.) or β-actin (A5541, Sigma) was used as a loading control. The relative intensities of protein bands were analyzed using the Image Lab™ analysis software from Bio-Rad Laboratories.

### Immunohistochemistry

Immunohistochemistry was performed using standard protocols. Briefly, cryostat coronal slices at the level of the midstriatum (Bregma 1.00) and substantia nigra pars compacta (SNpc) (Bregma − 3.20) were incubated with the primary antibody against sLeX, together with specific antibodies against ionized calcium binding adaptor molecule 1 (Iba-1, 019–19741, Waco Pure Chemicals), neuronal nuclei (NeuN, PA578639, Thermo Fisher Scientific Inc.) or tyrosine hydroxylase (TH, AB152, Millipore), followed by incubation with anti-mouse and anti-rabbit secondary antibodies conjugated to Alexa 488 and Alexa 568, respectively (Thermo Fisher Scientific Inc.). Nuclei were stained with DAPI, and slides were mounted with Ibidi mounting medium (Ibidi). Green, red and blue fluorescence of at least 10–15 random microscopic fields were acquired per sample using a Leica DMi8-CS inverted confocal microscope with Leica LAS X software. Fluorescence intensity was quantified with ImageJ software, normalized to the total area and expressed as fold change relative to control, using the ImageJ software analysis (National Institutes of Health). Control experiments for non-specific binding were performed in parallel by the omission of the primary antibodies.

### Total RNA isolation and qRT-PCR analysis

Total RNA, from mouse striatum, was extracted using the Izol-RNA lysis reagent (5PRIME), according to the manufacturer’s instructions. Afterwards, 1.5 µg of RNA from each sample was subjected to reverse transcription, using the SuperScript II reverse transcriptase (Thermo Fisher Scientific Inc.), with random hexamers. Real-Time PCR was performed using the SensiFAST™ SYBR® Hi-ROX kit (Bioline) in QuantStudio 7 Flex Real-Time PCR System (Applied Biosystems). Amplification of *Tnf-α*, C–C motif chemokine ligand 2 (*Ccl2*), *Fut7* and *Fut9* was performed using specific primers, described in Table 1, following the appropriate cycling program.

**Table 1:** Primer sequences for mouse gene expression analysis

**Gene**	**Forward primer (5′–3′)**	**Reverse primer (5′–3′)**
*Eef*	ACACGTAGATTCCGGCAAGT	AGGAGCCCTTTCCCATCTC
*Ccl2*	TCAGCCAGATGCAGTTAACG	GATCCTCTTGTAGCTCTCCAGC
*Tnf α*	GCCTCTTCTCATTCCTGCTTG	CTGATGAGAGGGAGGCCATT
*Fut7*	CAGATGCACCCTCTAGTACTCTGG	TGCACTGTCCTTCCACAACC
*Fut9*	ATCCAAGTGCCTTATGGCTTCT	TGCTCAGGGTTCCAGTTACTCA

The mRNA levels of target genes were normalized to the eukaryotic elongation factor 1-α (*Eef*) and expressed as fold change relative to control, using the ∆∆Ct method.

### Statistical analysis

All results are expressed as mean ± SEM values. Data were analyzed by one-way ANOVA, and differences between groups were determined by post hoc Bonferroni’s test, using GraphPad Prism 5.0 (San Diego, CA, USA). Student’s *t*-test was applied when two groups were analyzed. Means were considered statistically significant at a *p* value below 0.05.

## Results

### Markers of glycation in the MPTP model of PD

**Fig. 2:**
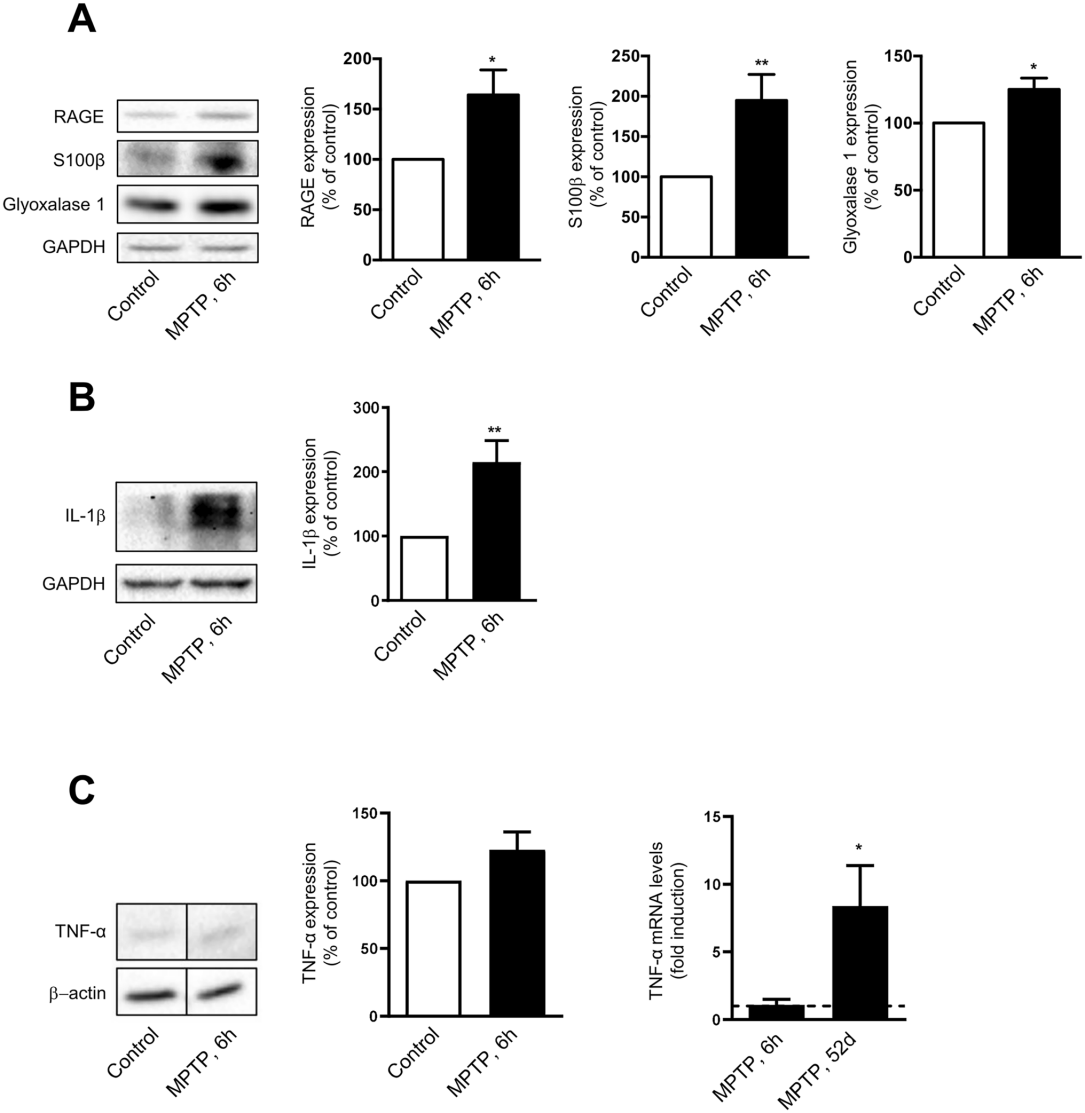
Effect of MPTP on the expression of glycation and neuroinflammation markers in mouse striatum. C57BL/6 mice treated with saline (control) or MPTP (40 mg/kg; single i.p. injection) were sacrificed 6 h or 52 days after MPTP administration, and striatum was immediately dissected. Striatal protein extracts were run in SDS-PAGE and immunoblotted for RAGE, S100β and glyoxalase 1 (**A**), IL-1β1 (**B**) and TNF- α (**C**). GAPDH or β-actin was used as a loading control. Results represent at least three independent experiments. Data are expressed as mean values ± SEM indicated as percentage of the respective controls. *p < 0.05 and **p < 0.01 vs control. **C** Total RNAs were analyzed by qRT-PCR using specific primers. The relative amount of *Tnf-α* transcripts were calculated using the ∆∆Ct method, normalized for the expression of the housekeeping gene *Eef*. Results represent three independent experiments. *p < 0.05 vs respective control

**Fig. 3:**
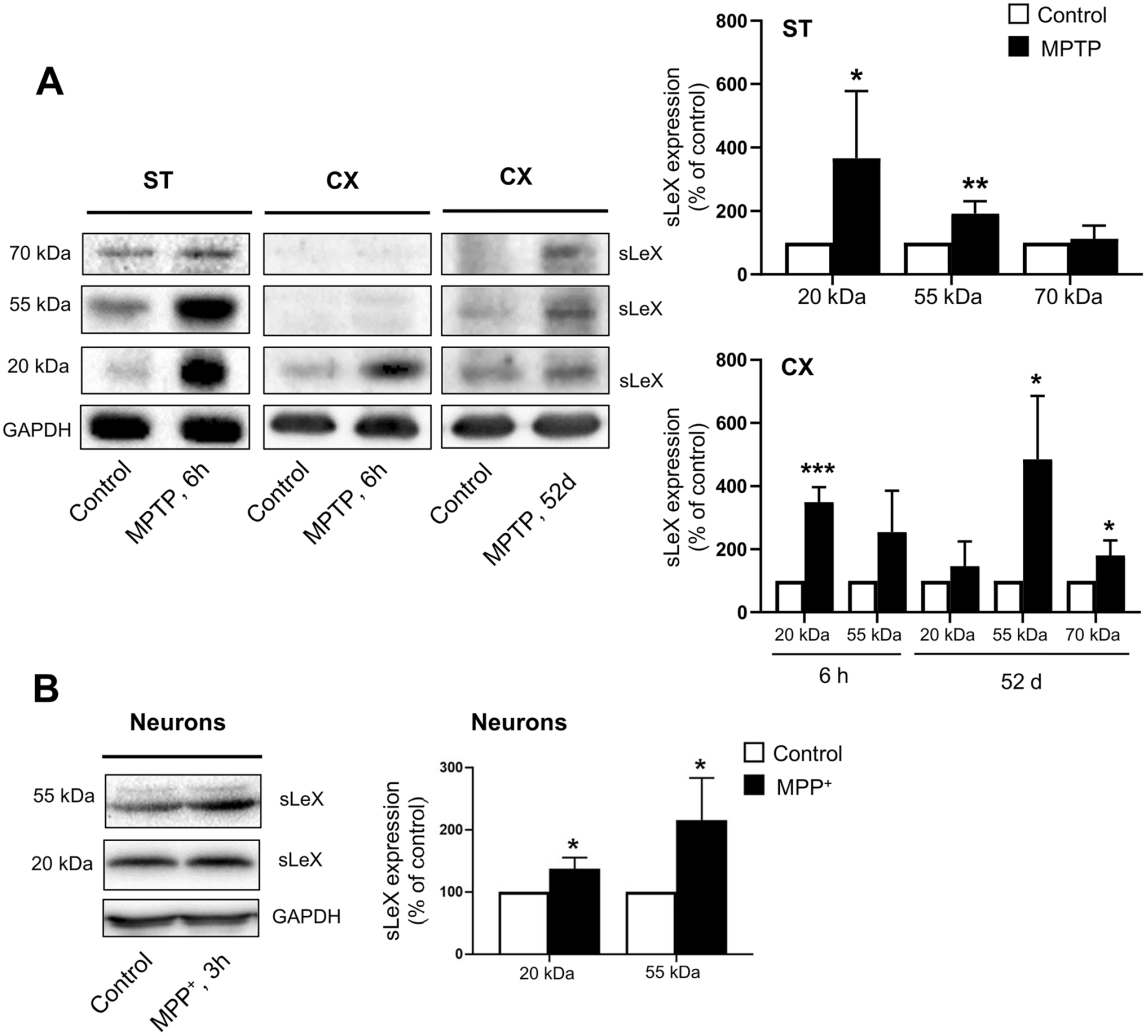
Expression of sLeX in the mouse brain and primary neurons in experimental models of PD. C57BL/6 mice were treated with saline (control) or MPTP (40 mg/kg; single i.p. injection). Mice were sacrificed 6 h or 52 days after MPTP administration, and striatum (ST) and cerebral cortex (CX) were immediately dissected. Primary cultures of C57BL/6 mouse cortical neurons (neurons), prepared as described in the “Material and methods” section, were treated with sterile Milli-Q water (control) or 0.5 mM MPP^+^ for 3 h. Protein extracts from mouse brain (**A**) or primary neurons (**B**) were run in SDS-PAGE and immunoblotted for sLeX. GAPDH was used as a loading control. Results represent at least three independent experiments. Data are expressed as the mean values ± SEM indicated as percentage of the respective controls. *p < 0.05, **p < 0.01 and ***p < 0.001 vs control

Increasing evidence supports oxidative stress and inflammation as critical driving forces in the pathology of PD. Receptor for advanced glycation end products (RAGE) is a multiligand receptor that has been implicated in several mechanisms underlying the pathogenesis of neurodegenerative diseases. Activation of RAGE by its ligands triggers rapid generation of reactive oxygen species (ROS) and inflammatory cytokine up-regulation through RAGE signal transduction and activation of transcription factors [23]. Increased levels of RAGE and its ligands were reported in PD patients [24, 25] and PD models [25]. Here, we found that at 6 h after MPTP treatment, there was a significant increase in the levels of RAGE, as well as one of its main ligands S100β (Fig. 2A). Importantly, MPTP-driven RAGE and S100β expression are accompanied by the up-regulation of glyoxalase 1 expression (Fig. 2A). This enzyme is a major deglycating enzyme that converts methylglyoxal (a byproduct of glycolysis) into lactoylglutathione, which is converted into D-lactate, reducing the formation of AGEs [26]. Therefore, glyoxalase may serve as an indicator of glycation in the striatum since its expression is driven by cellular compensatory mechanisms triggered upon MPTP administration. Since neuroinflammation is a hallmark of PD, we assessed if treatment with MPTP for 6 h also induced the pro-inflammatory marker cytokine interleukin-1β (IL-1β) and tumour necrosis factor-α (TNF-α). As shown in Fig. 2B and C, an increase in IL-1β is observed while no significant changes were detected in TNF-α levels, despite the tendency to increase, in the mouse striatum after MPTP treatment. Nevertheless, at 52 days post-MPTP there is an eightfold increase in the mRNA levels of this cytokine (Fig. 3C). We have recently described astrogliosis in this disease model [27]. Additionally, microgliosis is observed in the striatum of mice treated with MPTP for 6 h (Supplementary Fig. 1), evaluated by staining with the specific marker Iba-1. Concomitantly, at this time point, there was a sixfold increase in the mRNA levels of *Ccl2* (Supplementary Fig. 1), a chemokine released by activated microglia, that functions as a potent chemoattractant for monocytes and immune cells while playing an important role as a mediator of neuroinflammation [28]. These results clearly show that this treatment regimen with MPTP generates early inflammation that extends in time, which may trigger RAGE-dependent mechanisms in the striatum.

### Increased expression of sLeX in models of PD

sLeX is a glycan associated with inflammation and overexpressed in different pathological conditions [17, 18]. To explore the relevance of this glycan in PD, we evaluated sLeX expression in protein extracts from mouse striatum and cortex upon MPTP administration. Western blot analysis (Fig. 3A) showed distinct protein bands stained for sLeX, with approximate molecular weights of 70, 55 and 20 kDa, in mouse striatum, indicating the presence of different proteins decorated with sLeX. Importantly, 6 h after MPTP administration, the expression levels of sLeX-decorated proteins significantly increased in the striatum. The proteins bearing the sLeX epitope are differentially expressed in the striatum, since the relative abundance of the three molecular weight blot bands is different, being the 70-kDa band the less abundantly expressed and the only one that is not significantly increased by MPTP treatment. These differences in the relative protein abundance are maintained after MPTP administration.

Others and we have previously demonstrated the deleterious effects of MPTP, which are not exclusively confined to the nigrostriatal axis but could also be detected in the mouse cerebral cortex [5, 29]. Accordingly, analysis of mouse cortex revealed an up-regulation of sLeX-decorated proteins with the same approximate molecular weight as those detected in the striatum. Additionally, in the 6-h MPTP treatment, only the 20 kDa band is increased, whereas after long-term exposure (52 days), the levels of the 55 and 70 kDa sLeX bands become significantly elevated in the cortex (Fig. 3A). These results suggest that MPTP increases the levels of sLeX-decorated proteins very rapidly, while being differently activated in distinct brain compartments. The effect of MPTP in the striatum is faster and then progressively spreads into other brain regions such as the cortex.

To better assess the effect of MPTP on sLeX expression, we used primary mouse neurons treated with MPP^+^, the active metabolite of MPTP. sLeX is detected in primary neurons and its expression is significantly up-regulated by MPP^+^ treatment (Fig. 3B). Interestingly, as previously observed in brain tissue, neurons also express sLeX-decorated proteins of 20 and 55 kDa, which are up-regulated after MPP^+^ treatment. However, the 70-kDa immunoblot band was not detected under our experimental conditions, and the effect of MPP^+^ on sLeX expression in primary neurons is not as striking as in the mouse brain. Such differences may reflect that other cells besides neurons express sLeX in the brain and/or that neuron-neuron or neuron-glia interactions in the brain may contribute to increased sLeX expression in response to MPTP-induced neurotoxicity.

### Increased expression of FUT7 in models of PD

**Fig. 4:**
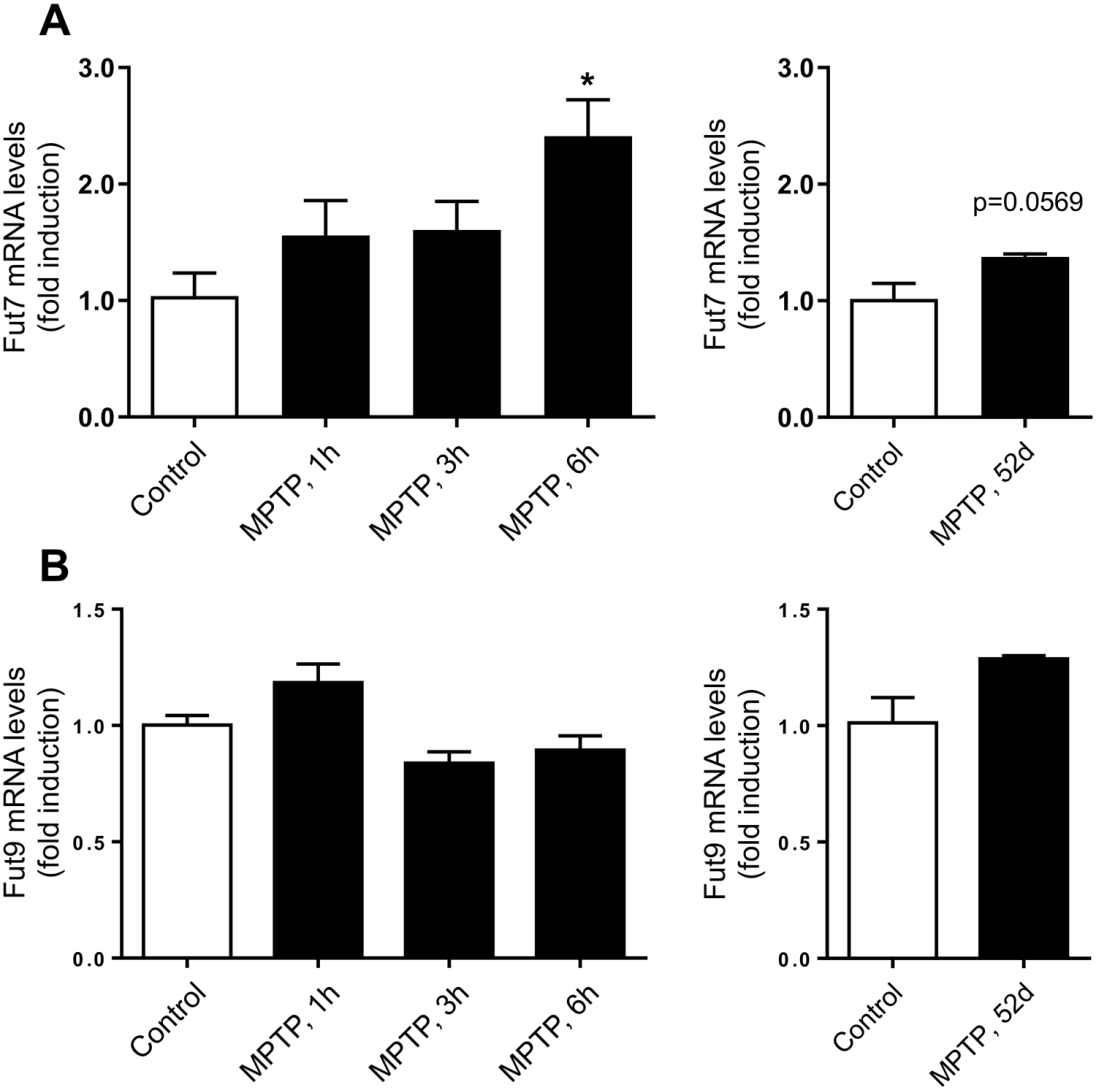
Effect of MPTP on the levels of Fut7 and Fut9 mRNA in mouse striatum. C57BL/6 mice treated with saline (control) or MPTP (40 mg/kg; single i.p. injection) were sacrificed 1 h, 3 h, 6 h or 52 days after MPTP administration, and striatum was immediately dissected. Total RNAs were analyzed by qRT-PCR using specific primers. The relative amounts of *Fut7* (**A**) and *Fut9* (**B**) products were calculated using the ∆∆Ct method, normalizing for the expression of the housekeeping gene *Eef*. Results represent three independent experiments. *p < 0.05 vs control

Since we observed an increase in sLeX-modified proteins in response to MPTP, that prompted us to evaluate the mRNA levels of FUT7, a rate-limiting enzyme in sLeX synthesis [12]. Indeed, a significant increase in Fut7 mRNA levels (Fig. 4A) was detected in the mouse striatum after 6 h of MPTP treatment. The increase in Fut7 mRNA levels in short-term treatments appears to be time-dependent, with a peak at 6 h post-MPTP, since after 52 days of treatment the values were not different from controls. Another fucosyltransferase highly expressed in the brain is FUT9, which catalyzes LeX and not sLeX biosynthesis and may compete for type 2 *N*-acetyllactosamine interfering with sLeX biosynthesis [30] (Fig. 1). No differences were observed in Fut9 mRNA levels after MPTP treatment (Fig. 4B). These results are in agreement with the increased levels of sLeX-decorated proteins found in the brain of MPTP-treated mice and suggest FUT7 up-regulation as the major responsible for the increased expression of sLeX driven by MPTP.

### sLeX expression in the brain is associated with neurons and microglia

**Fig. 5:**
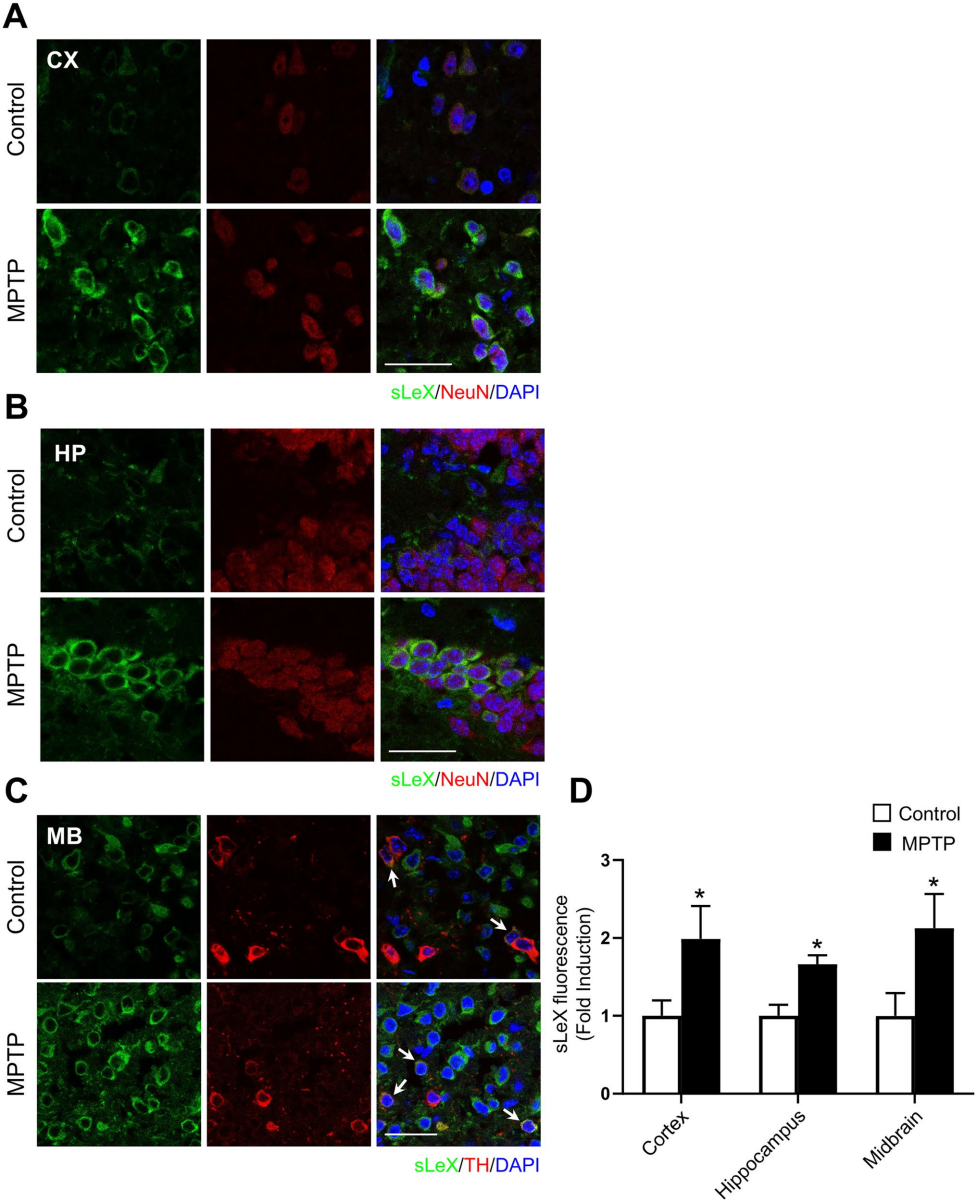
Effect of MPTP on the expression of sLeX in mouse brain. C57BL/6 mice treated with saline (control) or MPTP (40 mg/kg; single i.p. injection) were sacrificed 6 h after MPTP administration, and tissue was immediately processed for immunohistochemistry. Coronal brain sections were stained with a specific antibody anti-sLeX (CD15s) (green) plus anti-NeuN antibody (red) (**A** and **B**) or anti-TH (tyrosine hydroxylase) antibody (red) (**C**), and nuclei were stained with DAPI (blue). Microphotographs for cerebral cortex (CX) (**A**), hippocampus (HP) (**B**) and midbrain (MB) (**C**) represent at least three independent experiments. (**D**) Mean fluorescence intensity was quantified with ImageJ, and data are expressed as the mean values ± SEM, indicated as fold of the respective control. *p < 0.05 vs control. Scale bar 50 µm. Merged panel and arrows show co-localization

The in situ sLeX expression in response to MPTP treatment was further evaluated by immunohistochemistry analysis. As expected, sLeX was detected in the mouse cortex, and the expression of this glycan was significantly increased upon MPTP administration (Fig. 5A). Additionally, cells carrying this carbohydrate epitope were also found in the hippocampus (Fig. 5B) and the midbrain (Fig. 5C), and in all the analyzed brain regions, MPTP administration significantly increases sLeX expression (Fig. 5D). Additionally, most cells expressing sLeX in the brain are neurons, as demonstrated by the co-localization of NeuN and sLeX staining (Fig. 5A and B). Interestingly, we also found that sLeX is expressed in TH-positive neurons (dopaminergic neurons) in the substantia nigra (Fig. 5C), although only a small proportion of TH-positive cells carry the sLeX epitope.

These data confirm that, as showed by our immunoblot results, sLeX expression is significantly up-regulated in response to MPTP intoxication, and its distribution in the brain is wide. Moreover, they show that in the brain, the surface expression of sLeX is cell-type specific, since only particular groups of cells express this glycan in different brain regions.

**Fig. 6:**
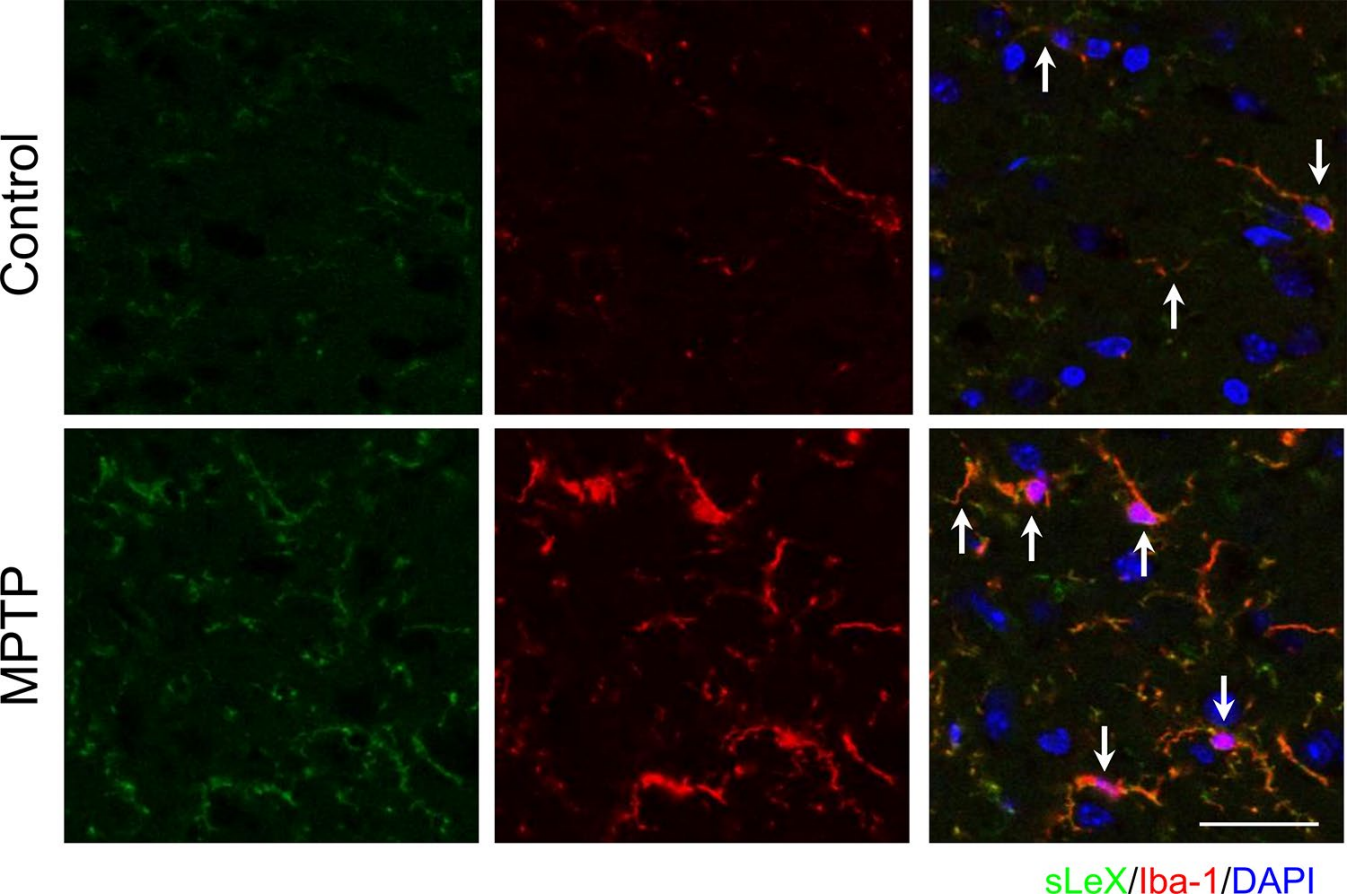
Identification of microglia expressing sLeX in mouse striatum. C57BL/6 mice treated with saline (control) or MPTP (40 mg/kg; single i.p. injection) were sacrificed 6 after MPTP administration, and tissue was immediately processed for immunohistochemistry. Coronal brain sections were stained with a specific antibody anti-sLeX (CD15s) (green), microglia were stained with anti-Iba-1 antibody (red), and nuclei were stained with DAPI (blue). Scale bar 50 µm. Merged panel and arrows show co-localization

The co-localization of sLeX and Iba-1, a microglia marker (Fig. 6), suggests microglia also express sLeX in the striatum of both controls and MPTP-treated animals. In addition, no sLeX was found in GFAP-positive cells (data not shown), indicating that in these experimental conditions, astrocytes do not carry surface proteins with this specific modification.

Altogether, our results show that sLeX is found broadly in mouse brain, and its expression is responsive to MPTP treatment. Although sLeX is found in microglia, it is predominantly expressed in neurons where its levels increase upon MPTP exposure.

## Discussion

The study of the glycome is complex, and the importance of aberrant glycosylation in neurodegenerative diseases is being increasingly recognized due to the emergence of novel analytical methods and more sensitive tools. In particular, the characterization of protein glycosylation in PD is still poorly explored. Thus, here we aimed to identify alterations in the expression of the sialofucosylated glycan, sLeX, in PD mouse brain.

We have previously demonstrated that sub-acute MPTP administration in mice causes motor impairment, depletion of nigral dopaminergic neurons, oxidative stress, mitochondrial dysfunction and neuroinflammation compatible with PD pathophysiology [4, 5, 31, 32]. Here, we complement the characterization of this PD model and demonstrate that MPTP induces the up-regulation of RAGE, a multiligand receptor of the immunoglobulin superfamily [33], most likely as a consequence of increased oxidative stress in the brain. Over-expression and activation of RAGE produce vicious cycles that perpetuate oxidative stress and contribute to neuroinflammation through nuclear factor-Bκ (NF-κB)-driven up-regulation of genes involved in the inflammatory response [23]. Concomitantly, we identified the up-regulation of the proinflammatory cytokines IL-1β and TNF-*α*. RAGE activation depends on several ligands, and one of the most important is S100β [34]. S100β is up-regulated in several neuropathological conditions, including PD, and drives excessive RAGE activation that culminates in ROS production, gliosis and neurotoxicity [35]. Accordingly, we found significantly increased S100β expression in the brain of the mouse PD model. Overall, our results are in accordance to previous observations showing increased expression of RAGE in PD patients’ brains and demonstrating the role of this receptor in neuronal dopaminergic degeneration in different mouse models of PD [25, 33, 34]. Although AGE formation is spontaneous and unavoidable, active protective mechanisms are involved in abrogating its toxic effects. The glyoxalase system is the most relevant catabolic route of various glycating agents [36]. Interestingly, we found that MPTP administration leads to the up-regulation of glyoxalase 1 expression. Glyoxalase 1 is under regulation of the transcription factor nuclear factor erythroid 2–related factor 2 (Nrf2) [37], which we previously showed to be activated in cell and animal models of PD [31, 38]. Glyoxalase 1 up-regulation may constitute an early compensatory neuroprotective mechanism upon MPTP administration to counterbalance the toxic insult.

Glycosylation is one of the most important modifications of proteins and lipids, and cell surface glycoconjugates are thought to play essential roles in various biological functions. Proper glycosylation is vital for brain function, and recently, aberrant glycosylation has been implicated in neurological disorders [7, 8], but little is known about the role of glycans in PD.

Glycosylation is affected by the NF-κB and Nrf2 signalling pathways [39, 40], which in turn have an important role on inflammation in PD, and in PD models [3–5, 41]. Indeed, pro-inflammatory cytokines have been shown to modulate cell surface glycosylation by regulating the expression of key enzymes, namely glycosyltransferases and sulfotransferases, that catalyze the biosynthesis of glycan chains, inducing the expression of specific carbohydrates at the cell surface [16]. Therefore, in this study we hypothesized that these pathways could modulate the glycosylation pattern in the brain.

One of the glycans directly up-regulated by inflammation in cancer and other diseases is sLeX [11, 14, 42]. Here, we demonstrate for the first time that MPTP increases the expression of sLeX in a time-dependent manner, as soon as 6 h after MPTP administration, in mouse striatum and cortex. sLeX increase occurs concomitantly with the previously identified increase in the levels of IL-1β (Fig. 2A), and other mediators of inflammation (Fig. 2C and Supplementary Fig. 1) and oxidative stress in the PD murine brain [4, 32].

In this study, we found increased levels of Fut7 mRNA in the striatum of MPTP-treated mouse. Although we were able to detect Fut9 mRNA in the same brain region, we did not find a significant modulation in its mRNA levels nor in the levels of LeX (Supplementary Fig. 2), suggesting no up-regulation of LeX by MPTP. In the experimental conditions used, Fut4 mRNA levels were undetectable (data not shown). Together, these results reinforce the conclusion that the up-regulation of sLeX is a specific response to the MPTP-triggered insult. The increase in Fut7 expression occurs early after MPTP administration, possibly contributing to the increased sLeX expression observed in mouse brain. Nevertheless, it cannot be excluded the contribution of other mechanisms for the observed increase in sLeX expression, such as increased expression of the carrier proteins or enzymes involved in upstream steps of the glycan biosynthesis, or even increased expression of sLeX on the glycolipids on the plasma membrane of the cells.

Pro-inflammatory cytokines such as IL-1β, IL-6 and TNF-α have been shown to modulate the expression of glycosyltransferases and sLeX cell surface expression [11, 16]. Interestingly, these pro-inflammatory mediators are also increased in human PD and experimental disease models, as shown here and in previous works [4, 43], which reinforces the importance of our results. The activated NF-κB and Nrf2 signalling pathways likely modulate both FUT7 and cytokine expression.

It has been previously described that sLeX is expressed in white blood cells and in microglia [11, 20]. Here, we confirmed that sLeX is expressed at the cell surface of microglia, but notably, it is mainly expressed in neurons, including a sub-population of nigral dopaminergic neurons. Importantly, sLeX expression in response to MPTP is not confined to the nigrostriatal axis but is also evident in other brain regions, including the cortex and hippocampus. The identification of sLeX in neurons has never been described before, and further work, which is not within the scope of this study, is needed to specifically characterize its biological importance.

In cancer cells, others and we showed that a higher activity of FUTs and aberrant sLeX expression are associated with enhanced metastatic activity [17, 42]. sLeX up-regulates cancer cell survival pathways and inflammatory cytokines, increases proliferation, adhesion, migration and expression of trophic factors [44]. Interestingly, our preliminary results (Supplementary Fig. 3) suggest that inhibition of FUTs with 2-fluorofucose [45], and thus sLeX synthesis, protects neurons from MPP^+^ toxicity. Thus, we hypothesize that sLeX may have distinct roles in different cells, triggering cell death pathways and abnormal cell–cell contacts in neurons, whereas in cancer cells, it up-regulates survival pathways, proliferation, adhesion and migration. More work is needed to better characterize the role of sLeX in this disease.

FUT3, FUT5, FUT6 and FUT7 are members of the human α(1,3)-FUT family known to be involved in sLeX biosynthesis. Whereas FUT7 is expressed in both rodents and humans, FUT3, FUT5 and FUT6 genes are not present in the rodent genome [12]. Nonetheless, the N-linked glycosylation sites are very similar among human and mouse proteins, and glycoproteins from human brain show a similar profile of brain-specific N-glycans as glycoproteins from mouse brain [46, 47], although some differences may exist between human and mouse on O-linked sites [47]. Glycan molecules are identical in mouse and human tissues, so it is feasible to assume that identical glycoproteins decorated with sLeX will similarly impact the function/activation of surrounding cells. We have previous robust data showing neuropathological parameters triggered by this dose of administration of MPTP that resemble most of the neuropathological features of human PD, including increased oxidative stress and mitochondrial dysfunction, astrogliosis and microgliosis, death of dopaminergic neurons and severe motor symptoms [4, 5, 31, 32]. Thus, the alteration of sLeX biosynthesis and presentation due to MPTP treatment in mouse brain should reflect, at least partially, the role of this glycan in human PD.

The importance of proper levels of fucosylated glycans in the brain is reinforced by the fact that patients with congenital disorders of glycosylation with impaired fucosylation show evident neurological phenotypes [48]. In particular, SLC35C1-CDG IIc patients, carrying mutations in the GDP-fucose transporter, which limits the biosynthesis of sLeX and other fucosylated glycans, display moderate to severe intellectual disability and microcephaly [49, 50].

The underlying mechanism that drives increased sLeX epitope on proteins and the functional importance of this glycan in the brain are still unknown, but our data suggest that the sLeX role in the brain goes beyond leukocyte extravasation and may include signalling and immunomodulation.

### Supplementary Information

Below is the link to the electronic supplementary material.

Supplementary file1 (PDF 177 KB)

## Data Availability

Not applicable.

## Abbreviation

Iba-1: Ionized calcium binding adaptor molecule 1
IL-1β: Interleukin-1β
FUT: Fucosyltransferase
LeX: Lewis X
MPP^+^: 1-Methyl-4-phenylpyridinium iodide
MPTP: 1-Methyl-4-phenyl-1,2,3,6-tetrahydropyridine
NeuN: Neuronal nuclei
PD: Parkinson’s disease
RAGE: Receptor for advanced glycation end products
ROS: Reactive oxygen species
sLeX: Sialyl Lewis X
TH: Tyrosine hydroxylase
TNF-α: Tumor necrosis factor-α
