# Supplementary material for: Altered expression of Sialyl Lewis X in experimental models of Parkinson’s disease
Source: J Mol Med (Berl). 2024 Jan 10;102(3):365–77. doi: 10.1007/s00109-023-02415-3 (PMC10879467; doi:10.1007/s00109-023-02415-3)
Supplement: Supplementary file 1 — Supplementary file1 (PDF 177 KB) [file 109_2023_2415_MOESM1_ESM.pdf]

Supplementary Figure 1

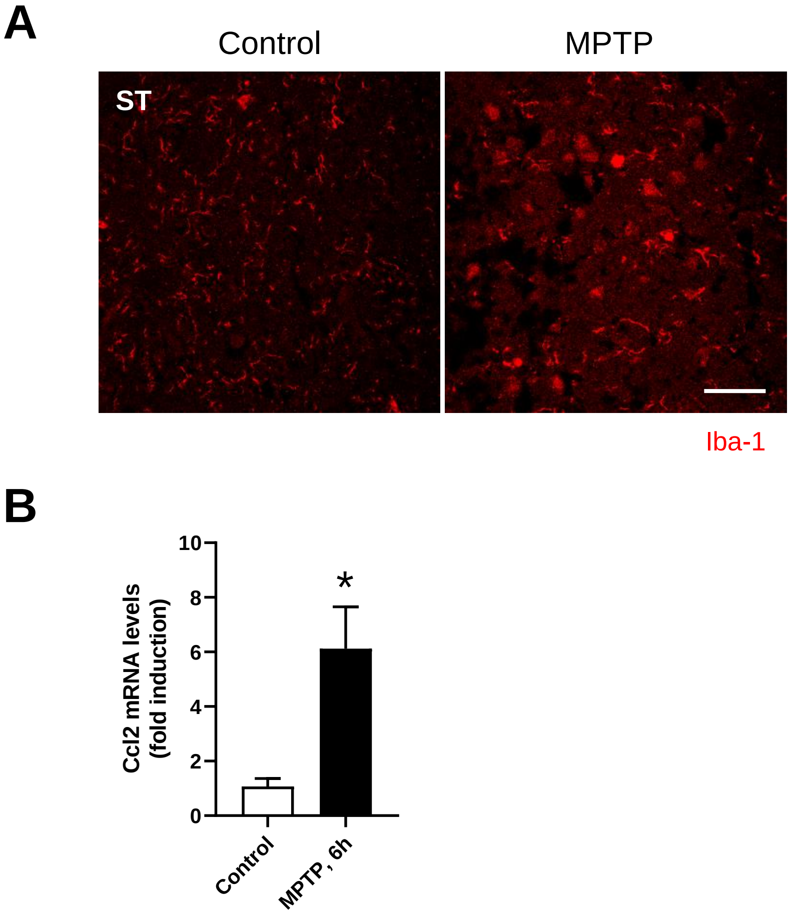

Supplementary Figure 1 – Induction of inflammation in the striatum of MPTP-treated mice. C57BL/6 wild-type mice were i.p. injected with MPTP (40 mg/Kg) and sacrificed 6h post-treatment. **A)** Microglia was identified by Iba-1 immunostaining in coronal sections at the level of striatum (ST - Bregma 1.00) from control and MPTP treated mice. All confocal immunofluorescence images were taken using the same laser power, gain and objective. Photomicrographs shown are representative of three independent experiments with at least three animals per group. Scale bar = 40 µm. **B)** Total RNAs were analyzed by qRT-PCR using specific primers. The relative amounts of C-C motif chemokine ligand 2 mRNA (*Ccl2*) were calculated using the ΔΔCt method, normalized for the expression of the housekeeping gene Eef. Results represent three independent experiments. *p<0.05 vs control.

Supplementary Figure 2

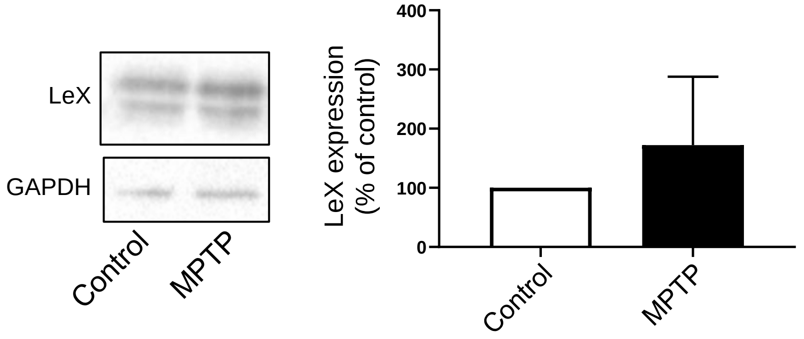

Supplementary Figure 2 – Effect of MPTP on Lewis X expression in mouse striatum. C57BL/6 mice were treated with saline (Control) or MPTP (40 mg/Kg; single i.p. injection). Mice were sacrificed 6h after MPTP administration, and striatum was immediately dissected. Striatal protein extracts were run in SDS-PAGE and immunoblotted for Lewis X (LeX) (Hibridome SH1). GAPDH was used as an endogenous control of loading. Results represent at least three independent experiments. Data are expressed as the mean values ± SEM indicated as percentage of the respective controls.

Supplementary Figure 3

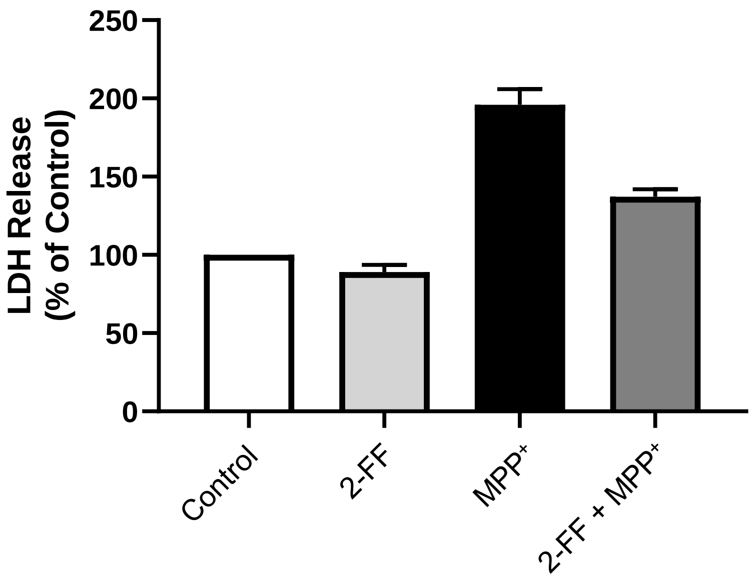

Supplementary Figure 3 – Effect of 2-FF on MPP+-induced neuronal cell death. C57BL/6 mouse cortical primary neurons culture (15 DIV) were pre-treated with either vehicle or 1mM 2-FF (2-Fluorofucose) for 7 days, and afterwards cells were treated with or without 250 μM MPP+ for 16 h. 2-FF is a cell-permeable fluorinated fucose derivative that blocks the fucosylation and subsequent sLeX biosynthesis, previously shown to be effective in cells and in vivo in mice.

Cell death was assessed by the amount of LDH released to the cell culture supernatant. Cell death is expressed in percentage of LDH release in control neurons. Results are expressed as percentage of control. Data shown are values of three technical replicates.
